# Accessing community health services: challenges faced by poor people with disabilities in a rural community in South Africa

**DOI:** 10.4102/ajod.v1i1.19

**Published:** 2012-10-10

**Authors:** Lisbet Grut, Gubela Mji, Stine Hellum Braathen, Benedicte Ingstad

**Affiliations:** 1 Department of Health Research, SINTEF, Norway; 2 Centre for Rehabilitation Studies, Stellenbosch University, South Africa; 3 Department of Psychology, Stellenbosch University, South Africa; 4 Department of Community Medicine, University of Oslo, Norway

## Abstract

Poor people with disabilities who live in poor rural societies experience unique problems in accessing health services. Their situation is influenced by multiple factors which unfold and interplay throughout the person’s life course. The difficulties do not only affect the person with a disability and his or her family, but also impact on the relevant care unit. The barriers are rooted in a life in poverty, upheld and maintained by poverty-reinforcing social forces of the past and the present, and reinforced by the lack of the person’s perspective of the health services. This article explores how difficulties may interact and influence access to and utilisation of health services, and how this may render health services out of reach even when they are available. The study reveals that non-compliance is not necessarily about neglect but could as well be a matter of lived poverty. The study was based on in-depth interviews with people with disabilities and family members, and semi-structured interviews with health personnel. The data analysis is contextual and interpretive. When offering health services to people with disabilities living in resource-poor settings, services should take into consideration the person’s history, the needs, and the resources and abilities of the family group. Rethinking access to health services should transcend a narrow medical institutionalisation of health professional’s training, and include a patient’s perspective and a social vision in understanding and practice. Such rethinking requires health service models that integrate the skills of health professionals with the skills of disabled people and their family members. Such skills lie dormant at community level, and need to be recognised and utilised.

## Introduction

South-East of Mthatha, towards the rugged coast of the Indian Ocean, lies a beautiful landscape of steep hills and deep valleys. This is Amathole district in the Eastern Cape, South Africa. In the rainy season the hills are green, the fields yield their crops and the valley beds flow in rapid streams towards the ocean. In the dry season everything turns yellow and barren. Narrow gravel roads, sometimes only walking trails, connect the clusters of rondavels and small houses that are mostly located on the hilltops and high mountain sides. This is the home of Mthunzi (a pseudonym), a young man of twenty-one. Mthunzi has epilepsy which has led to physical and intellectual impairment. He lives in a small rondavel together with his mother and father, spending his days sitting outside the hut. He does not talk and pays little or no attention to what happens around him. He is not paralysed but he does not walk. Except for protecting him from the sunshine and the rain, the parents leave him alone. He does not get medication for his condition and has minimum follow-up from the health services. The local community health worker described Mthunzi as a case of neglect, where the parents are neglecting their son. Why do they do nothing to help him? How could their lack of effort be understood? This case is from a fieldwork conducted in Amathole district, South Africa. The case illustrates the intertwined relationship between disability, poverty, and access to health services in a resource poor setting. In order to understand situations, such as the one illustrated in the case, we have applied a broad approach exploring how a person’s life circumstances are influenced by a variety of social, cultural and political factors throughout a person’s life course. In this article we will explore how the many difficulties in the life of a person living in a resource-poor environment may influence access to and utilisation of health services, and how this may render health services out of reach even when they are available.

### Poverty, disability and health services

Health is one of the fundamental rights of every human being, and a robust and accessible health system is necessary to fulfil this right (WHO 2008a). There is a close relationship between poverty, ill-health and limitations in access to health services (WHO 2010a). It is widely recognised that poverty extends beyond deprivation of income and material assets, and that poverty reflects deficiency in many dimensions in a person’s life (Townsend 2006, Green 2006). Poverty is also connected to lack of opportunity to lead a healthy life (Fukuda-Parr 2006). In this respect, health services, along with food, water, sanitation, and knowledge and education, can be considered necessary conditions for good health. Thus, poverty and reduced access to good quality health services are intertwined (Wagstaff 2002). In order to alleviate poverty and contribute to better lives for people who are poor it is necessary to address this complexity and focus on the interconnectedness between the many elements of poverty and health (World Bank 2000, Yeo 2006:74).

People in poor countries have less access to health services than those in better-off countries, and within countries poor people have less access to health services than better-off citizens (Peters *et al*. 2008). Furthermore, lack of visible improvement after therapy may hamper usability, and cultural belief systems may colour the decisions on health matters as much as modern knowledge of health, disease and treatment (Kleinman 1980, Ross 2008). People with disabilities are particularly vulnerable in this regard, and their situation is further exacerbated in poor, rural areas (Iezzoni, Killen & O’Day 2006, Daley *et al*. 2011). Despite variations, people with disabilities are among the most vulnerable and marginalised groups of any population, and they often face exclusion from mainstream health services (Ingstad & Whyte 2007:1, Saloojee *et al*. 2007, Loeb *et al*. 2008, Smith *et al*. 2004, Rohleder *et al*. 2009, McColl, Jarzynowska & Shortt 2010). Among the factors which reduce access to health services for poor people with disabilities are unavailability and inaccessibility of the health services, combined with financial constraints and ignorance of available services, inadequate and inaccessible transport (Saloojee *et al.* 2007, Van Rooy *et al*. 2012).

Africa has the greatest disease burden of any continent and the poorest health services (WHO 2008b, Oestergaard *et al*. 2011, Gore *et al*. 2011). The African region carries one quarter of the world’s burden of illness, only 3% of the global health work force, and less than 1% of the world’s financial resources (WHO 2006). The World Health Report *Health systems financing: the path to universal coverage* (WHO 2010b) highlights a worldwide shortage of almost 4.3 million doctors, midwives, nurses and support workers. Thirty-six of the 57 countries with severe shortages are in Africa.

Seen in an African context South Africa has a fairly well developed health system. Health policies were amongst the first policies to be given attention when the ANC-led democratic government came to power in 1994 (Barron & Roma-Readon 2008:vii). The new government focused on fundamental improvements in basic infrastructure, as well as poverty alleviation strategies. Funding was reallocated from specialised services in large hospitals towards decentralised health services, and primary health care facilities and community health care were emphasised (Gray and Clarke 2000, Magnussen, Ehiri, and Jolly 2004, Tshabalala-Msimang 2003).

In a South African context poverty is linked to past history where apartheid played a major role, but also to the uneven distribution of wealth and welfare in today’s society (Crais 2002).

Despite the relative wealth of South Africa compared to other countries in the region, poverty is still rampant for a large majority of the population, and universal and equitable access to health care is still out of reach. The rural areas are the poorest, most under-served and historically most neglected (Barron & Roma-Readon 2008). In 2001 seven of the 10 poorest municipalities in South Africa were located in the Eastern Cape (HSRC 2004). Almost 40% of the South African population live in rural areas, but at a macro level the population pattern in South Africa, as in many other countries world wide, shows increasing urbanisation. However, there is evidence of cycling migration by bidirectional streams of people between the urban and rural categories (Statistics South Africa 2006).

In the face of widespread poverty, social grants have become the main source of income and livelihood for many families. When a child is born at the hospital he or she gets a birth certificate and a health card. These documents are necessary to access social grants, such as child support grant or disability grant. More than 1.1 million out of approximately 50 million South Africans currently receive disability grants (Statistics South Africa 2007), and in the Eastern Cape about 74% of people with disabilities receive such grants (Jelsma *et al*. 2008). While this money may prevent complete destitution for individuals and families, it is not enough to escape poverty completely (Surender *et al*. 2007, Maistry and Vasi 2010). However, studies show that if disability grants are combined with other grants, such as old age pensions and child support grants, these grants may provide rural households with sufficient money to prevent dire poverty (Hansen & Sait 2011:93).

#### The study setting – rural South Africa

The study setting is Madwaleni hospital catchment area in Amathole district in the Eastern Cape Province, which is one of the poorest, most highly populated and most neglected rural areas in South Africa (Statistics South Africa 2007). Poor infrastructure still expose people residing in the area to a variety of health hazards. Lack of clean water and good sanitation create a number of health threats, including epilepsy caused by the tapeworm Taenia solium, most commonly found in fecally contaminated water and undercooked pork (Veary & Manton 2008). Other common diseases in Amathole district are tuberculosis, HIV and AIDS. People with disabilities are considered to be particularly vulnerable (Rohleder *et al*. 2009) both because they may lack knowledge about how to protect themselves, and because information about the diseases may be neither available nor accessible to them (Groce 2005).

Madwaleni hospital catchment area was chosen because it is one of the areas targeted for the new South African Government’s Health Care Reform. The district’s health services are based at Madwaleni District Hospital, a 200-bed secondary hospital. Up to 2007 the hospital paid no specific attention to community based services. However, with the introduction of community health care services and their policy for health and rehabilitation, a professional rehabilitation team joined the hospital’s medical team. This team started community and home visits to locate people with disabilities living in the area as well as disability and rehabilitation awareness raising programmes. Eight clinics have been built around Madwaleni District Hospital as a result of the implementation of primary health care services. The eight clinics are managed by skilled nurses and are staffed by nurses and community health workers (CHWs). The CHWs are primarily young people trained in basic home care skills. Doctors and rehabilitation professionals visit the clinics monthly. The hospital and its eight clinics face the challenge of staff shortages and high staff turnover, especially with regard to medical and rehabilitation professionals.

## Research methods and design

We conducted qualitative in-depth interviews in the Madwaleni Hospital catchment area. The interviews were done in November 2008. Informants were identified by the rehabilitation personnel at Madwaleni District Hospital, by health workers at the local health clinics, by local chiefs and through snow-balling. Interviews were conducted with 24 persons with a disability and/or their family members, fourteen men and boys, ten women and girls. The informants represent a variety of life situations, types of disabilities, ages, family conditions and settlements (Kvale 1996). Included in the sample are people with physical, sensory and intellectual impairments. Caretakers were interviewed as proxies for the seven children below 18 years and the seven adults who could not speak for themselves. However, the children and adults attended the interviews and were encouraged to participate in the conversation.

The study also includes six semi-structured interviews with professionals at Madwaleni District Hospital, seven interviews with professional health workers, and five with unskilled health workers at four local health clinics.

The interview guide was developed from an interview guide used in similar studies in Yemen and Kenya (Grut & Ingstad 2006) and was adapted to the South African setting. The study was conducted as a joint effort between the authors of this article who are social scientists from Norway and South Africa. All interviews were done by the authors. We split into two teams consisting of one Norwegian and one South African researcher in each team. The interviews were done in the informants’ homes. The language used was the informant’s mother tongue, in most cases Xhosa, with a simultaneous translation into English. The notes which were taken during the interviews were transcribed and discussed among the team members every afternoon during the field work. Ethical clearance was obtained from ethical research committees in Norway and South Africa. Consent was obtained from all informants.

### Data analysis

The data was analysed according to a contextual and interpretive perspective (Denzin & Lincoln 2005), which is well suited when the aim is to explore the multidimetional relationship between disability and access to and usability of health services. Within this perspective the person’s life history is analysed according to a life course approach (Elder and Giele 2009). Understanding how a person’s life is shaped by social, cultural, and historical forces will add to the understanding of the person’s situation today

## Ethical consideration

The project is approved by the ethical committee of Stellenbosch University South Africa and the Norwegian Social Science Data Services - the body that approve all research in Norway involving individual data.

## Results

This study shows that access to health services for people with disabilities who live in resource poor communities is influenced by multiple factors which unfold and interplay throughout the person’s life course. The difficulties do not only affect the person with a disability and his or her family, but also impact on the relevant care unit. This complex relationship will be discussed through the case of Mthunzi and his parents, presented in the vignette of this paper. The family was introduced to the researchers by a health worker at one of the local health clinics. The health worker told us that Mthunzi had a bad health condition, and that the parents had declined the health worker’s invitations to come to the clinic. According to the health worker, the parents had been encouraged many times by the health personnel to bring Mthunzi to the local health clinic, but had declined. One community health worker said that their decline was a sign of non-compliance and neglect. We were curious to learn more about the family’s situation and to understand the underlying reasons for their choices.

Visiting the parents, we learned that Mthunzi had been without medication for the epileptic seizures for years. At the time of our visit he had five to six seizures a day. Years with lack of medication has obviously caused severe intellectual and physical impairment. He easily became tens, and reacted with fits and aggression to disturbances and changes in his environment. To avoid this, the parents disturbed him as little as possible. The interview with the parents revealed that the family had faced many difficulties throughout the years, rooted in social, cultural, and historical forces. The interplay between the many challenges which have unfolded throughout the years, have kept them in poverty and limited their access to health care services.

### Past experiences with health services influence today’s expectations

Mthunzi’s parents explained how they had tried everything to help their son. Mthunzi’s mother remembered that he was a healthy child up to three years of age. He then got pains in his back and bones. The mother took him to the District Hospital, which at that time neither had rehabilitation facilities, nor local health clinics or outreach services. It took a whole day by foot to get to the hospital, but at that time Mthunzi was little and she was younger and much stronger than she is now. The mother told us that they had kept him hospitalised for a year and a half, and that he developed epilepsy during the stay. The parents could not remember what the doctors at the hospital did to him. Neither did they know why he got sick or why he developed epilepsy. They could not remember if they got any explanation about his condition. When he was discharged, the doctors prescribed epilepsy medication and the parents were told to come to the hospital regularly for supplies. The parents expected the medication to eliminate the seizures. When this did not happen, they soon stopped going to the hospital for more tablets, and as a result Mthunzi has been without epilepsy medication since early childhood.

#### The search for other options

When the medication from the hospital did not work, they took him to a traditional healer. The healer told them that Mthunzi had been bewitched by a close relative, and suggested methods for how they should relate to this relative in order to make the seizures stop. This created a feeling of despair in the parents because they had a good relationship with this relative, and they did nothing about it. Shortly after, they were hit by another unfortunate incidence when lightning struck their house and the house burned down. These series of misfortune raised a feeling of hopelessness, and they gave up seeking treatment for Mthunzi’s condition.

#### Poor infrastructure - a barrier to local health services

When the local health clinics were established in the area this could have represented new opportunities for Mthunzi and his parents. But in order to receive health services, they have to turn up at the clinic. They explained that they live four hours walk on narrow gravel roads from the nearest health clinic. The roads are steep and in some places there are only paths to climb up and down. There is no access to safe drinking water or latrines in the neighbourhood, and there is no electricity in the area. Public transport in the form of small pickup-trucks is available on the main roads. Because Mthunzi must be accompanied, transport implies paying for an extra person. Wheelchairs are only exceptionally admitted on public transport, and also only when charged an extra fee. A person who does not behave in a socially acceptable manner will often not be admitted on the bus. When Mthunzi has been transported in the past it has resulted in him having seizures, soiling himself and becoming violent. This situation is embarrassing, arduous, and frightening to the parents, and as a result they opt not to use public transport. The only alternative is to transport him in a wheelbarrow. However, the parents are elderly and not physically strong. The distance is too far for them to walk and the hills are too steep for them to manage with a wheelbarrow.

#### The loss of a regular income

The family is poor and without any regular income. Because of the impoverished landscape (Crais 2002), families in Amathole rarely make an income from what they grow. Many of the men in this area have had to stay away from their families for long periods, sometimes for years, in order to find work. Labour migration may represent an opportunity by providing a sustainable livelihood when there is no opportunity at home (Narain 2006). But migration also deprives the household of a family member who could otherwise contribute to the daily chores. In the past Mthunzi’s father used to be away for long periods working in the mines in Johannesburg. When he was a miner, the father had a regular income and the family managed reasonably well compared to the general standard of living in the area. At that time the family had some cows and some sheep. However, the father was injured some years ago and he lost his job. He has been unemployed since then. Because he has not received any compensation from the mining company, the lack of a regular income has forced them to sell the live stock one by one in order to manage. At the time of this field work they had three sheep left.

A disability grant for Mthunzi could have been an income option for this family, and it could have provided them with means to pay for some of the expenses connected to accessing health services. As Mthunzi was born at home he was never registered at the hospital’s birth clinic. Because of this he has no Identity-card and consequently he is not entitled to a disability grant. Similar to Mthunzi many adults and elderly do not have a grant because they were born at home. To obtain a grant as an adult or elderly person one must go through procedures which imply several visits to public offices and health services in order to collect and fill in the necessary documents before one can go to the office and collect the grant. Mthunzi’s parents are not capable of bringing Mthunzi through these procedures.

## Discussion

Our case illustrates how people with disabilities who live in resource-poor areas experience a number of barriers associated with individual and societal poverty. Their situation houses problems of poverty and suffering (Farmer 2001) that stem from constraints created by the interplay between individual misfortune, an impoverished landscape, and the constraints set by the politics of the past and present (Crais 2002). Through an exploration of how their lives have been influenced by a variety of social, cultural and political barriers it is possible to understand the rationale behind their choice of not seeking health care even when the needs are obvious and services are available in the area.

Health professionals and patients carry different positions and obligations in relation to the health system, and they base their expectations and actions on very different perspectives. However, in order for health services to meet a person’s needs, and offer adequate and adapted services, it is fundamental to understand the rationale for the person’s actions and priorities. This is particularly important in an impoverished setting. To incorporate the person’s life history into the case and to understand the person as a member of a family group and of a community under constant strain will provide a better understanding of the reasons behind non-compliance, and subsequently offer adapted services. The African philosophy of *Ubuntu* emphasises that ‘a person is a person through their relationship to others’ (Boon 1996, van Niekerk 2007). In this context the impacts of disability reach far beyond the disabled individual and affect the entire family, who become ‘the disabled family’ (Ingstad 1997). As the case in this study illustrates, understanding how a person’s situation is influenced by individual (mis)fortune and changing social, cultural, and historical forces would reveal that non-compliance is not necessarily about neglect. Non-compliance could as well be a matter of lived poverty throughout one’s life course. One aspect of poverty is the struggle of the individual and the family to cope and make a life for themselves despite hardships.

The case in this study was chosen because it illustrates significant findings of the overall study. The informants first and foremost tried to improve their life situation within the limits that are set by social, cultural, and political forces. For Mthunzi’s family this implied improving his situation by having the epileptic seizures and aggressive behaviour controlled. Mthunzi’s parents had learned that the best way to avoid seizures was to leave him in peace and not take him away from home. Similar to many other informants in this study, the parents of Mthunzi saw little use in making the effort to bring him to the clinic for medication. The effort involved in moving him was not worth the gain of getting a medication that the parents had perceived as having no sustainable effect. In fact, the journey might cause them embarrassment and danger by him having a fit of rage and soiling himself. Given their situation, this non-compliance could be understood as a rational choice. The chance of receiving help from the health professionals was weighed against the money spent and the effort and social costs involved.

Similar to many of the informants in this study the parents of Mthunzi lacked knowledge on how to acquire and sustain good health as well as knowledge about training and rehabilitation. In particular they lacked knowledge on how medical treatment works. Basically, they lacked the means to respond to the initiatives of the local health personnel. Similar to many of the other informants, they all too often had to choose between high costs - use of scarce money, effort and energy - weighed against their daily chores and responsibilities. These choices were made against a background of earlier experiences, (lack of) knowledge on how to sustain and gain good health, unfulfilled expectations of a cure, and how health, illness and (mis)fortune are understood and coped with within their cultural context. Faced with the burden of poverty, lack of compliance could be understood as a rational choice against a background of a social suffering with deep historic roots.

## Conclusions

Poor people’s choices and decisions must be understood as shaped by the many negative factors that influence their lives. The complexity of the barriers which unfold throughout a person’s life course create difficult situations and may prevent the person from accessing health care services even when the services are available. Looking at each factor separately without understanding the connection between them can easily make us think that some of them are rather trivial. By taking a deeper look into the situation, it is evident that the interplay between the many different elements creates situations with significant obstacles. The combination of the many factors creates barriers to accessing health care services that may be too challenging to overcome even with well-functioning local based health care services in the area.

When offering health care services to people who live in resource-poor settings services should take into consideration the needs as well as the resources and abilities of the family group. Rethinking the notion of access to health services in under-resourced areas, calls for a need to transcend a narrow medical institutionalisation of health professional’s training, and to also include a social element in their understanding and practice. Further, in line with an extended health professional approach there is a need to develop the training and the tasks of the local, often unskilled, health workers accordingly.

The close link between disability and poverty cannot be underplayed and should be acknowledged and integrated with health policy and strategies. The future development of health care services should focus on bringing medical services and treatment out to the disabled person and the families; with a stronger emphasis on involving skilled and specialised medical and health professionals higher up in the hierarchy in outreach and home-based services. In cases where this is not possible, there is a need to strengthen accessible transport facilities in order to bring the patient to the hospital.

When focusing solely on the limited resources in the health care services and the lack of compliance of the patient, there is a risk of ignoring the resources of the family and the community. A family perspective requires looking for innovative models that integrate the skills of health professionals with the contextual knowledge and individual experiences of disabled people and their family members. Such resources lie dormant at community level and should be recognised and utilised.

Health service systems are expected to respond to and implement health policies and strategies. In South Africa the introduction of local health clinics is one strategy to reach out to the poor population with health services. It is, however, a serious challenge to these initiatives that the realities of the poor population produce huge barriers to accessing the services and thus threaten health equity. While sufficient resources in health care is crucial, it is also key to improved access to health care that the knowledge and experiences of poor people are integrated into health services and utilized as a resource. This further brings into question whether individually based incentives, such as disability grants, may overshadow the overwhelming need for improvements in infrastructure, including roads, affordable and accessible transportation, and provision of safe drinking water and sanitation facilities. Not least, this study indicates that a community based approach to health and health services should acknowledge that a person’s life is influenced by a variety of social, cultural and political factors. This may be a viable strategy for incorporating the knowledge and experiences of poor communities, and for reaching out to the poorest of the poor. Further, improving the skills of the community health workers in basic health and rehabilitation could alleviate the load that parents of children with impairments experience.
